# NUP133 Controls Nuclear Pore Assembly, Transcriptome Composition, and Cytoskeleton Regulation in Podocytes

**DOI:** 10.3390/cells11081259

**Published:** 2022-04-07

**Authors:** Manuel Rogg, Jasmin I. Maier, Markus Ehle, Alena Sammarco, Oliver Schilling, Martin Werner, Christoph Schell

**Affiliations:** 1 Institute of Surgical Pathology, Faculty of Medicine, Medical Center-University of Freiburg, 79106 Freiburg, Germany; manuel.rogg@uniklinik-freiburg.de (M.R.); j.i.maier@gmx.de (J.I.M.); markus-ehle@outlook.com (M.E.); alena.sammarco@uniklinik-freiburg.de (A.S.); oliver.schilling@uniklinik-freiburg.de (O.S.); martin.werner@uniklinik-freiburg.de (M.W.); 2 Freiburg Institute for Advanced Studies (FRIAS), University of Freiburg, 79106 Freiburg, Germany

**Keywords:** NUP133, nuclear pore complex, nucleoporin, podocyte, SRNS, podocytopathy, nucleoporopathy

## Abstract

Steroid-resistant nephrotic syndrome (SRNS) frequently leads to end-stage renal disease, ultimately requiring kidney replacement therapies. SRNS is often caused by hereditary monogenic mutations, specifically affecting specialized epithelial cells (podocytes) of the glomerular filtration barrier. Mutations in several components of the nuclear pore complex, including NUP133 and NUP107, have been recently identified to cause hereditary SRNS. However, underlying pathomechanisms, eliciting podocyte-specific manifestations of these nucleoporopathies, remained largely elusive. Here, we generated an in vitro model of *NUP133*-linked nucleoporopathies using CRISPR/Cas9-mediated genome editing in human podocytes. Transcriptome, nuclear pore assembly, and cytoskeleton regulation of *NUP133* loss-of-function, mutant, and wild-type podocytes were analyzed. Loss of NUP133 translated into a disruption of the nuclear pore, alterations of the podocyte-specific transcriptome, and impaired cellular protrusion generation. Surprisingly, comparative analysis of the described SRNS-related *NUP133* mutations revealed only mild defects. Am impaired protein interaction in the Y-complex and decrease of NUP133 protein levels might be the primary and unifying consequence of mutant variants, leading to a partial loss-of-function phenotype and disease manifestation in susceptible cell types, such as podocytes.

## 1. Introduction

The kidney filtration barrier is essentially required for physiological homeostasis and composed of glomerular endothelial cells, the glomerular basement membrane, and podocytes—highly specialized epithelial cells [1,2]. Glomerular diseases affecting the glomerular filtration barrier (GFB) can ultimately result in chronic kidney disease (CKD) and are a major contributor to global CKD burden [3,4]. Podocytopathies are driving factors for the disruption of the glomerular filtration barrier and translate into impaired selectivity for macromolecules, resulting in proteinuria and nephrotic syndrome [5,6]. Steroid-resistant nephrotic syndrome (SRNS) is one entity of glomerular disease and often associated with hereditary monogenic mutations [7,8]. An increasing number, more than 50, of monogenetic hereditary podocytopathies causing SRNS have been identified so far [7,9]. One recently described group of mutated genes encodes for components of the nuclear pore complex (NPC). These NPC genes include *NUP85*, *NUP93*, *NUP107*, *NUP133*, *NUP160*, and *NUP205* [10,11,12,13,14].

The NPC forms a channel across the nuclear envelope, facilitating not only selective nucleocytoplasmic transport but also direct regulation of gene activity [15,16,17,18,19]. Around 30 different genes and more than 500 nuclear pore proteins, named nucleoporins (NUPs), constitute this multiprotein structure. The NPC is assembled by and composed of subcomplexes such as the Y-complex of the outer rings and inner ring complex. SRNS-causing mutations affect both, the inner ring complex via NUP93 and NUP205 as well as the Y-complex via NUP85, NUP107, NUP133, and NUP160. If not (congenital) lethal, mutations of essential “housekeeping” NUPs often cause complex genetic syndromes [20,21]. Intriguingly, some of these nucleoporopathies lead to very specific genotype–phenotype relations, where different NUPs, or even point mutations in the same NUP, affect different cell types [11,15]. One such example for cell type-specific phenotype manifestations of nucleoporopathies is SRNS. Specific genetic variants of the Y-complex NUPs *NUP107* and *NUP133* cause Galloway–Mowat Syndrome (GAMOS; SRNS and cerebral defects), isolated SRNS, or isolated gonadal dysgenesis without SRNS [11,12,13,14,22]. These observations rise questions about the functional consequences of these mutations and whether they are cell type-specific, e.g., for podocytes [15]. A better understanding of the underlying pathogenic mechanisms could potentially lead to novel personalized therapy approaches, as successfully exemplified for other entities of genetic SRNS [23,24,25,26].

In this study, we modeled the loss of *NUP133*, and SRNS-causing *NUP133* variants, in vitro to examine podocyte-specific structural, transcriptional, and functional consequences of nucleoporopathies.

## 2. Methods

### 2.1. Cell Culture

An immortalized human podocytes cell line, AB8/13, was obtained from Moin A. Saleem, Bristol University, UK [27]. Podocyte culture was essentially performed as previously described [28]. RPMI 1640 (Thermo Fisher Scientific Inc., Waltham, MA, USA, #61870036) cell growth medium was supplemented with 10% FCS (Merck KGaA, Darmstadt, Germany, #S0615), 5 mM HEPES (Thermo Fisher Scientific, #15630-056), 1:1000 non-essential amino acids (Thermo Fisher Scientific, #11140050), 10 µg/mL ITS (Merck, #11074547001), 100 µM Sodium-Pyruvate (Thermo Fisher Scientific, #11360-039), and penicillin–streptomycin (Thermo Fisher Scientific, #15140122). HEK293T/17 cells (ATCC, Manassas, VA, USA, CRL-11268) were cultured in DMEM medium containing glulamine (Thermo Fisher Scientific, #31966-021) and supplemented with 10% FCS. Negativity for mycoplasma contamination was confirmed applying a mycoplasma PCR detection kit (Hiss Diagnostics GmbH, Freiburg, Germany) and Hoechst 33258 staining.

### 2.2. CRISPR/Cas9

For the generation of *NUP133* knockout podocytes, the CRISPR/Cas9 technique was used, as described before [28,29,30]. Several independent monoclonal clones were obtained from the polyclonal parental podocyte cell line and validated for the expression of podocyte markers and growth characteristics. Two of these clones were selected as background cell lines for genome editing. The following sgRNAs, targeting *NUP133* or *NUP107*, were used: *NUP133* sgRNA 1: 5′- CGGTTGATACCTGAGAGCTC(AGG)-3′, *NUP133 s*gRNA-2: 5′- GGCGTAGCTCGCTAAGCTCG(CGG)-3′, and *NUP107 s*gRNA: 5′- GGAATCCATATAGACGCATT(TGG)-3′. The sequence used for *NUP133* sgRNA-2 was described before by Braun et. al. [11]. *NUP133* gRNAs were inserted into a TLCV2 plasmid (Addgene plasmid #87360; http://n2t.net/addgene:87360 (last accessed 4 April 2022); RRID:Addgene_87360). *NUP133*-WT cell lines were created using the TLCV2 plasmid, without guide sequences. HEK293T cells were used for lentivirus production. After infection with lentivirus particles, podocytes were singled and grown on 96-well culture plates. The resulting clones were screened for loss of NUP133 expression, and one clone per sgRNA was selected for further validation and analysis.

### 2.3. Expression Plasmids

cDNA sequences coding for *NUP133* (RC204410, OriGene Technologies) and *NUP107* (SC126878, OriGene Technologies Inc., Rockville, MD, USA) were cloned into n-terminal FLAG epitope-tagged pcDNA6 and pWPXLD plasmids (Addgene plasmid #12258; http://n2t.net/addgene:12258 (last accessed 4 April 2022); RRID:Addgene 12258). Causative *NUP133* variants c.2922T>G (“Mutation-1”) and c.3335-11T>A (“Mutation-2”) were introduced using the Q5 site-directed mutagenesis kit (E0552S, New England Biolabs), according to the suppliers instructions. HEK293T cells were used for lentivirus production, and WT podocytes were subsequently transduced using these particles.

### 2.4. Immunoprecipitatio

cDNA constructs were expressed in HEK293T cells, and immunoprecipitation was performed using established protocols [28,31]. Briefly, HEK293T cells were transfected with the indicated pcDNA6 cDNA plasmids using the PEI (Polyethylenimin) method. After 24 h, cells were lysed in Triton-X-100 lysis buffer (1% Triton X-100, 20 mM Tris-HCL, 50 mM NaCl, 50 mM NaF, 15 mM Na_4_P_2_O_7_, 1 mM EDTA in ddH_2_O, pH 7.4), with protease inhibitors (#5892970001, Merck) and 1 mM Na_3_VO_4_ on ice for 30 min. Lysates were cleared by centrifugation (16,000× *g*, 4 °C, 15 min) and anti-FLAG M2 affinity agarose gel (#A2220, Merck) was added and incubated on an overhead shaker for 1 h at 4 °C. Subsequently, anti-FLAG M2 affinity agarose gels were sedimented by centrifugation and repetitively washed with Triton-X-100 lysis buffer. Bound proteins were eluted in × Laemmli buffer with DTT and denatured at 95 °C for 5 min. Lysates were analyzed by standard SDS-PAGE.

### 2.5. Western Blot Analysis

All steps for cell lysis were performed at 4 °C. Podocyte lysates were generated using RIPA buffer for 15 min. Subsequently, lysates were centrifuged at 16,000× *g* for another 15 min. After centrifugation, supernatants were mixed with a 2× Laemmli sample buffer containing DTT and denatured at 95 °C for 5 min. Protein concentration was determined by applying the Pierce BCA protein assay kit (#23225, Thermo Fisher Scientific). Finally, cell lysates were analyzed by SDS-polyacrylamide gel electrophoresis (SDS-PAGE) and western blotting applying the trans-blot turbo transfer system (Bio-Rad Laboratories, Inc., Hercules, CA, USA) and PVDF membranes (#1704157, Bio-Rad). Membranes were blocked in 5% BSA in TBST, and primary antibodies were incubated on membranes at 4 °C for 24 h. After washing in TBST, HRP-linked secondary antibodies were used for detection of primary antibodies using the HRP-ECL chemiluminescence reaction (#32109, Thermo Fisher Scientific) and an Intas chemiluminescence imager. Fiji ImageJ v1.52 was used for densitometry of western blot band intensities from independent replicates. Densitometries were expressed as the ratio of signal-to-TUBA band intensity per sample. For graphical presentation, values were normalized to the mean NUP107/TUBA, PPME1/TUBA, or COL18A1/TUBA ratio, respectively. These normalized ratios, per sample and individual experiment, were used for statistical analysis and are presented in the respective figures.

### 2.6. Antibodies and Fluorescent Dyes

The following antibodies were used for Western blot (WB) and/or immunofluorescence (IF): NUP133 (sc-376763, Santa Cruz Biotechnology Inc., Dallas, TX, USA, WB 1:2000, IF 1:300), NUP107 (SAB2702098, Merck, WB 1:1000, IF 1:200), nuclear pore complex proteins mAb414 (ab24609, Abcam, Cambridge, UK, WB 1:1000, IF 1:200), beta-Actin (4970S, Cell Signaling Technology Inc., Danvers, MA, USA, IF 1:400), VCL (HPA063777, Atlas Antibodies AB, Bromma, Sweden, WB 1:1000), TUBA (T9026, Merck, WB 1:5000), FLAG M2 (F3165, Merck, WB 1:5000), PXN (610051, BD Biosciences, Franklin Lakes, NJ, USA, NU, 1:300), COL18A1 (ab275746, Abcam, WV 1:1000), PPME1 (sc-25278, Santa Cruz Biotechnology, WB 1:2000), Hoechst 33342, trihydrochloride, trihydrate (H3570, Thermo Fisher Scientific, IF 1:1000), and Alexa Fluor phalloidin 488/555 (Thermo Fisher Scientific, IF 1:200–1:750). For IF Alexa Fluor conjugated anti-mouse or anti-rabbit secondary antibodies (A-31572, A-21127, A21245, A31570, A28175, Thermo Fisher Scientific, IF 1:500) and WB HRP-linked, anti-mouse (goat anti-mouse immunoglobulins/HRP, P0447, Dako, WB 1:10,000) or anti-rabbit (goat anti-rabbit IgG, HRP-linked antibody, #7074, cell signaling, WB 1:1000) secondary antibodies were used.

### 2.7. Immunofluorescence

For immunofluorescence (IF) analysis, podocytes were seeded on collagen IV (Merck, #C5533)-coated glass coverslips or 8-well slides (Ibidi GmbH, Gräfelfing, Germany, #80822 and #80827) for 24 h. Podocytes were fixated in 4% PFA (Electron Microscopy Sciences, #15714-S) in PBS (Thermo Fischer, #10010023) for 20 min. Cells were washed (3 times with PBS), and permeabilization was performed using Triton X-100, at 0.1% dilution in PBS for 3 min. For staining of NUP107, NUP133, and mAb414 additional heat-induced antigen retrieval (HIAR), using pH 9 Tris-EDTA buffer, was performed at 90 °C for 40 min. Samples were washed (PBS) and blocked using BSA at 5% (*w*/*v*) dilution in PBS for 1 h at room temperature. Primary antibodies were diluted in blocking solution and incubated at 4 °C overnight. Coverslips were washed (3 times with PBS) and incubated with secondary antibodies (Alexa Fluor secondary antibodies, Thermo Fisher Scientific), then diluted in blocking buffer. dsDNA (cell nuclei) was co-stained with Hoechst 33342 (Thermo Fisher Scientific, #H3570). Samples were incubated with secondary antibodies and Hoechst 33342 at room temperature for 45 to 60 min.

### 2.8. Microscopy

For immunofluorescence analysis an Axio Observer (Carl Zeiss AG, Oberkochen, Germany) microscope, equipped with the following components, was used: Colibri 7 light source, Axiocam 702 monochrome camera, ApoTome.2, microscope objectives for 10×/20×/40×/63×/100× magnifications, and fluorescence filter sets, i.e., 49 DAPI, 38 GFP, 43 HE dsRed, and 50 Cy5. Z-stack images were processed to maximum intensity projections (MIP) for presentation within the manuscript. The ZEN 3 software package was used (Carl Zeiss AG).

### 2.9. Nuclear IF Quantification

For the quantification of nuclear NUP133 and NUP107 IF signals, staining was performed as described. Immunofluorescence images were acquired by random sampling of nuclei in the Hoechst 33342 channel using a 20× objective. The QuPath v0.2.1 software (https://qupath.github.io/; last accessed 4 April 2022) was used for subsequent image analysis, and cell nuclei were segmented using the nucleus detection tool for Hoechst 33342 labeling of nuclei [32]. Inadequate segmented nuclei were manually corrected. Finally, mean fluorescence intensities per nucleus were measured, and nuclei were classified as NUP133 positive or negative, based on the threshold intensities validated by KO and WT cells. For analysis of nuclear NUP107 intensity in *NUP133* rescue or mutant cell lines, only NUP133 positive cells were included. For graphical presentation, nuclear intensities were normalized to the mean mAb414, NU107, or NUP133 intensity of all analyzed nuclei, respectively. A minimum of 145 nuclei, per cell line/genotype, were analyzed. For analysis of nucleus area and morphology, images from Hoechst 33342-labeled nuclei were segmented by thresholding and analyzed with Fiji ImageJ v1.52.

### 2.10. Integrin Adhesion Complex Analysis

Analysis of integrin adhesion complexes (IACs) was previously described [29,30]. Cells were seeded on 8-well µ-Slides (#80822, Ibidi GmbH) coated with collagen IV. Paxillin was used as marker to label adhesions by immunofluorescence staining. Fiji ImageJ was used for macro-based segmentation and analysis of IACs, as previously described [29,33]. Briefly, single IACs per cell were segmented and morphometric parameters of segmented IAC were measured. Measurements of corresponding cell areas were based on co-staining with phalloidin and IAC density (IAC number per cell area), per cell, and calculated. IACs of 30 podocytes per genotype were analyzed, and data were presented as mean per clone.

### 2.11. Cell Spreading Assay

Cell spreading analysis of podocytes was previously described [31]. Briefly, cells were detached by applying trypsin, washed in cell growth medium (3-times), counted, and diluted. For equilibration, floating podocytes and collagen IV-coated 8-well polymer coverslips (#80822, Ibidi GmbH) were incubated with cell growth medium in the incubator at 37 °C. After equilibration, cells were spread on 8-well coverslips for 30 min. After carefully washing with PBS (1 mM CaCl^2+^ MgCl^2+^), cells were fixed and permeabilized as described above and fluorescence stained using phalloidin (#A22283, Thermo Fisher Scientific). Fiji ImageJ v1.52 thresholding and object analysis tools were used for analysis. More than 100 cells, per genotype and replicate, were analyzed.

### 2.12. RNA Sequencing

One day before mRNA preparation, cells were seeded on collagen IV (C5533, Merck)-coated cell culture plates. Podocytes were washed by and scraped into PBS and pelleted by centrifugation. RNA was isolated from cell pellets using the Monarch Total RNA Miniprep kit (T2010S, New England Biolabs Inc., Ipswich, MA, USA), including gDNA depletion by gDNA binding columns and on column DNase-1 digest, according to the suppliers instructions. RNA quality number (RQN) was measured as 10.0 for all samples using an Agilent Fragment Analyzer system. Illumina 2 × 150 bp paired-end mRNA sequencing (NovaSeq 6000), including Poly(A) selection and library preparation, was performed by the GENEWIZ Germany GmbH. Two independent experiments were conducted: a first set of samples comparing *NUP133* WT and KO podocytes on two different monoclonal background (WT-1, WT-2, KO-1, and KO-2; two replicates each, total of eight samples) and second set comparing *NUP133* WT versus Mutant-1 and Mutant-2, expressed on background of the *NUP133* KO-1 cell line (three replicates each, total of nine samples). The NEBNext Ultra II Directional RNA Library Prep kit was used for experimental set-1, and NEBNext Ultra II RNA Library Prep kit (New England Biolabs) was used for set-2; bcl2fastq v2.19 was used for demultiplexing and adapter trimming. Per analyzed sample, more than 18 million reads were obtained. For further transcriptome and differential gene expression (DE) analysis, the Galaxy Europe bioinformatics platform was used [34]. The following tools and genomes were applied: Human Dec. 2013 (GRCh38/hg38) genome, Cutadapt or fastp [35], HISAT2 [36], featureCounts [37], and DESeq2 [38]. *NUP133* WT (2× WT-1, 2× WT-2) and KO (2× KO-1, 2× KO-2) clones were pooled for statistical analysis, and adjusted *p*-values were calculated using DESeq2. For analysis of mutant variants (three replicates per genotype), multi-comparison analysis was performed, and adjusted *p*-values were calculated using DESeq2.

The GenePattern platform was used for GSEA (gene set enrichment analysis, v20.2.4.) using all genes detected in at least one sample [39]. Parameters and GSEA results for the reactome pathway and GO-Term analysis are collectively presented in the Supplementary Dataset S1. GSEA of reactome pathways was imported into Cytoscape v3.7.2. using the enrichment map application [40,41]. A network of significant enriched gene sets (nominal *p*-value < 0.01 and FRD *q*-value < 0.25) was created by classifying gene sets with an overlap of >50% of mapped genes as interacting. A list of in vivo podocyte-specific expressed genes was derived from the available proteome [28,42] and transcriptome [31] analyses, as previously described [31]. In brief, genes were defined as a podocyte, specifically expressed (compared to non-podocyte glomerular cells) with significant enrichment in transcriptome and proteome datasets, as well as an expression level of >50 transcripts per million (TPM) in transcriptome analysis. For a detailed definition of cut-off values, see the Supplementary Dataset S1. To create a map of podocyte-specific protein–protein interactions (PPIs), significantly regulated and podocyte-specific expressed genes were used as seeds, in order to retrieve PPIs for these proteins from the BioGrid [43] database via Cytoscape’s built-in import tool. These PPIs were filtered for in vivo podocyte expressed genes (TPM > 50) to generate a subnetwork of possibly influenced PPIs in podocytes. The DAVID bioinformatics resource v6.8 was applied for enrichment analysis of specific overrepresented interactors in this network (Supplementary Dataset S1) [44]. Consensus adhesome components and podocyte small RhoGTPase proximity interactors were retrieved from previous published datasets (Supplementary Dataset S1) [31,45].

Sequencing data have been deposited in the NCBI Gene Expression Omnibus and are accessible via GEO series accession numbers GSE189703 and GSE189709 (differential expression analysis is enclosed in Supplementary Datasets S1 and S2).

### 2.13. 3D Structure Model

Structural model of the human nucleoporin NUP107–NUP133 interaction complex (PDB doi: 10.2210/pdb3CQC/pdb) modeling, NUP107 aa667 to aa924 and NUP133 aa932 to aa1156 (aa—amino acid), was created using previously published crystal structure data and the Mol*Viewer web app [46,47].

### 2.14. Statistical Analysis

GraphPad Prism 8 for graph preparation and statistical testing. Data are presented as mean  ±  S.E.M. Scatterplots indicate individual samples/cells/replicates, as stated in the figure legends, and are presented as used for statistical testing. Based on experimental design and data distribution, the following statistical tests were applied: unpaired Students *t*-test, unpaired *t*-test with Welch’s correction, one-way ANOVA with Dunnett’s multiple comparisons test, Kruskal-Wallis test with Dunnett’s multiple comparisons test, one-way ANOVA with Tukey’s multiple comparisons test, and one-way ANOVA with Sidak’s multiple comparisons test. Statistical significance was defined as *p* < 0.05, and numerical values are stated in the respective Figure panels (n. s.—non-significant). The number of independent experiments and analyzed cells or samples are stated in the methods section, figures, and/or figure legends.

## 3. Results

### 3.1. Knockout of NUP133 Impedes Nuclear Pore Complex Formation in Podocytes

**Figure 1:**
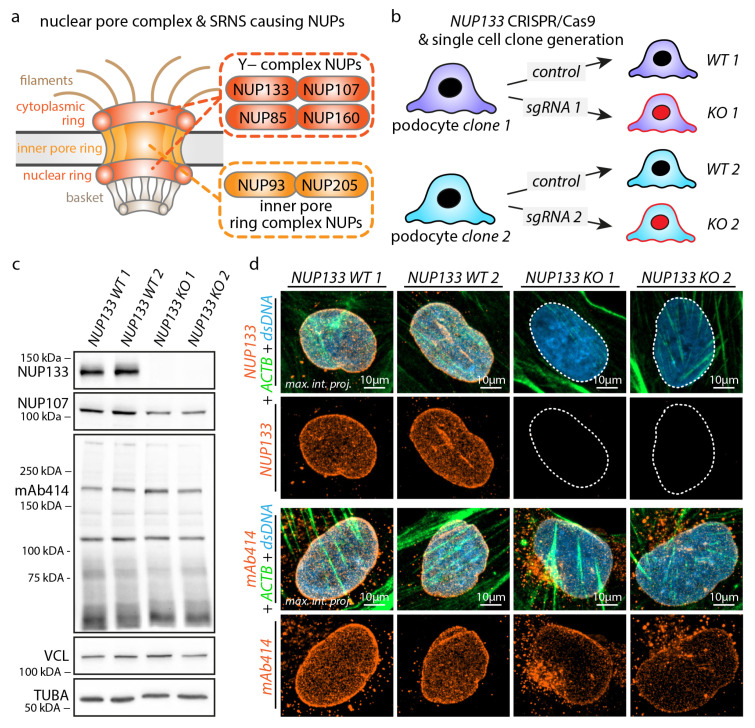
Knockout of *NUP133* impairs nuclear pore complex formation in podocytes. (**a**) Schematic of the nuclear pore complex (NPC) and nuclear pore proteins (NUPs), with known causative mutations for steroid-resistant nephrotic syndrome (SRNS). (**b**) Schematic illustrating the generation of two independent monoclonal *NUP133* knockout (KO) podocyte cell lines by CRISPR/Cas9 genome editing. (**c**) Western blot analysis confirmed the loss of *NUP133* in knockout cell lines and demonstrates the partial degradation of the Y-complex nuclear pore protein NUP107. In contrast, mAb414 (recognizing NUP358, NUP214, NUP153, and NUP62) [48,49] detected no altered protein levels. Vinculin (VCL) and alpha-tubulin (TUBA) served as loading controls. (**d**) Immunofluorescence analysis of mature NPCs in the nuclear envelope, by NUP133 and mAb414, confirmed loss of NUP133 expression and strongly reduced formation of mAb414 positive NPCs in the nuclear envelope. Concomitant increase of mAb414 positive vesicles in the cytoplasm was observed. Nuclei (dsDNA) were co-stained by Hoechst 33342 and whole cells by beta-Actin (ACTB). Dotted lines indicate the nuclear circumference; maximum intensity projections of z-stacks from whole nuclei are shown.

To dissect the specific impact of NPC disruption on podocyte biology, we employed the deletion of the Y-complex nucleoporin NUP133. The formation of the Y-complex is an essential prerequisite for the assembly of functional NPCs in the nuclear envelope and hindered by SRNS-causing mutations in *NUP107* and *NUP133* (Figure 1a) [11,15]. To model this proposed loss-of-function mechanism of *NUP133* variants [11,12], we generated two independent monoclonal *NUP133* knockout cell lines (KO-1 and KO-2) and matching wild-type control (WT-1 and WT-2) cell lines using CRISPR/Cas9-based genome editing (Figure 1b–d). NUP107 forms a physical interaction with NUP133, essentially required for proper assembly of the Y-complex [46]. For that reason, we analyzed NUP107 protein levels in *NUP133* KO podocytes and observed reduced NUP107 protein levels (Figure 1c and Figure S1). Based on this observation, we aimed to analyze the assembly of functional NPCs on a global level. The mAb414 antibody recognizes FG-repeats containing NUPs, such as NUP358, NUP214, NUP153, and NUP62, and is commonly employed to detect and visualize matured NPCs [48,49]. Using mAb414 for immunofluorescence staining did not demonstrate altered expression levels of FG-repeat NUPs or nuclear morphology, but greatly reduced assembly (−37% to −40% nuclear mAb414 fluorescence intensity) of mature NPCs in the nuclear envelope (Figure 1c,d and Figure S1). Interestingly, despite the absence of NUP133, some NPCs seemed still to be assembled in the nuclear envelope, as revealed by mAb414 staining. Therefore, we conclude that the deletion of NUP133 impedes (but not completely abolishes) the formation of mature NPCs in podocytes and represents a suitable model to study cell type-specific loss-of-function effects.

### 3.2. Causative NUP133 Mutants Restore Y-Complex and NPC Assembly

**Figure 2:**
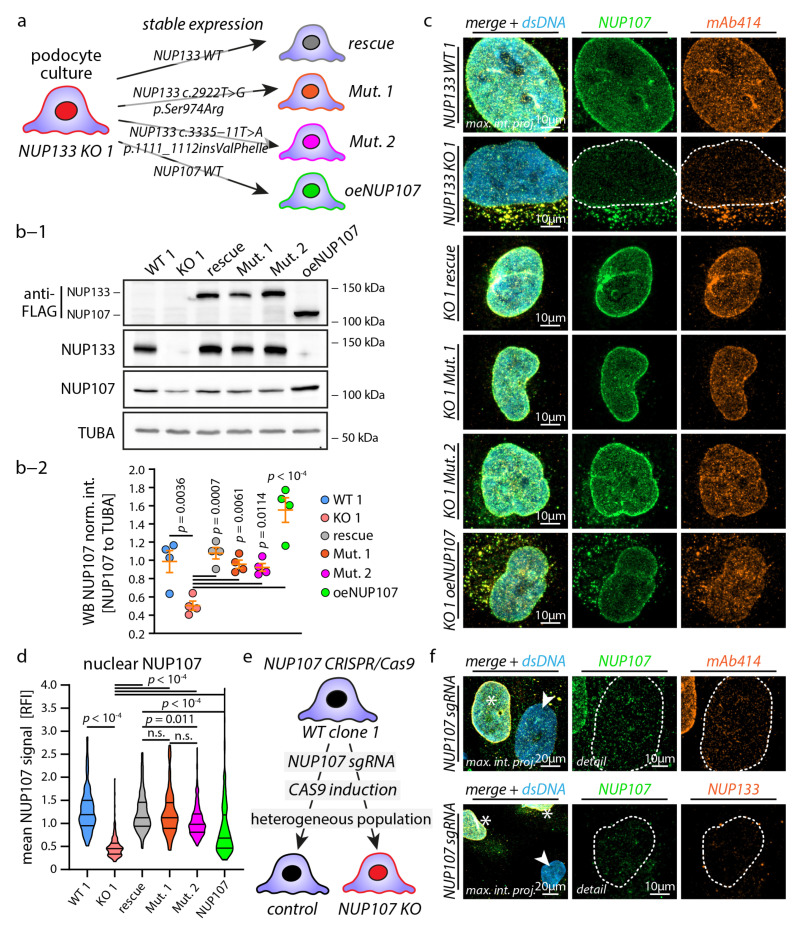
Causative NUP133 mutants restore Y-complex and NPC assembly. (**a**) Schematic depicting the generation of *NUP133* WT (rescue), *NUP133* mutant, and NUP107 overexpression (oeNUP107) cell lines by lentiviral transduction of *NUP133* KO-1 podocytes. (**b-1**) Western blot analysis confirms (re-)expression of FLAG-tagged NUP133 or NUP107 protein in KO podocytes and restored protein levels of endogenous NUP107. Alpha-tubulin (TUBA) was used as loading control. (**b-2**) The NUP107-to-TUBA western blot band densities of four independent replicates were analyzed (scatterplot). (**c**,**d**) Immunofluorescence analysis of mature NPCs in the nuclear envelope by NUP107 and mAb414 staining (nuclei were co-stained by Hoechst 33342; dotted lines indicate the nuclear circumference; maximum intensity projections of z-stacks from whole nuclei are shown). Nuclear NUP107 staining signals were quantified by measurements of relative fluorescence intensities (RFI) from at least 147 cells, per genotype, from one representative experiment, and expressed as violin plots. (**e**,**f**) Schematic illustrating the generation of an inducible *NUP107* KO podocyte population by CRISPR/Cas9 genome editing. Immunofluorescence analysis of this heterogeneous population of *NUP107* KO podocytes by NUP107, NUP133, and mAb414 demonstrated severely impaired formation of NPCs in KO cells (white asterisks indicate cells with preserved and white arrowheads with lost NUP107 expression; dotted lines indicate the nuclear circumference; maximum intensity projections of z-stacks from whole nuclei are shown; n. s.—non-significant).

After successful establishment of a *NUP133* loss-of-function in vitro model, we aimed to extend our model on SRNS-causing *NUP133* mutations. Consequently, we re-expressed *NUP133* wild-type (“NUP133-rescue”), *NUP133 c.2922T>G* (p.Ser974Arg) (“Mutant-1”), or *NUP133 c.3335-11T>A* (p.1111_1112insValPheIle) (“Mutant-2”) variants on the background of the *NUP133* KO-1 cell line (Figure 2a,b-1,b-2; see Figure S2 for structural localization of variants; respective mutations have been recently described) [11,12].

*NUP133* mutation variant-1, replacing Ser974 by Arg, is located within the NUP107–NUP133 interaction interface and impairs the binding of NUP133 to NUP107, as reported before (Figure S2) [11]. NUP133 mutation variant-2 affects the splice side between exons 25 and 26 of the *NUP133* gene, causing the insertion of three amino acids (Val-Phe-Ile) in an residue c-terminally located of the NUP107–NUP133 interaction interface, also affecting the binding of NUP133 to NUP107 (Figure S2) [12]. Since the functional deletion of NUP133 translates into partial NUP107 degradation (Figure 1c), we aimed to dissect the contribution of NUP107 to the *NUP133* KO phenotype by overexpressing *NUP107* (“oeNUP107”) in KO-1 podocytes. Given the sensitivity of NUP107 on compromised NUP133 function, we analyzed the functional impact of mutants on NUP107 protein level and NPC localization in the nuclear envelope (Figure 2b–d). Surprisingly, *NUP133* mutants were able to restore NUP107 distribution and nuclear localization, to an extent comparable to wild-type *NUP133*. Furthermore, staining for NPCs using mAb414 showed reestablished assembly of NPCs in the nuclear envelope (Figure 2c). However, detailed analysis showed a slight, but significant, reduction of nuclear NUP107 localization in *NUP133* mutant-2, compared to *NUP133* rescue cells (Figure 2d and Figure S1). Noticeably, both mutant variants exhibited a significantly reduced recruitment to the nuclear envelope, compared to wild-type *NUP133* (Figure S1). Altogether, these experiments indicate that reported causative *NUP133* variants present only mild structural phenotypes of the matured NPC, when compared to a complete *NUP133* knockout condition. Additional analysis of *NUP107* overexpression in *NUP133* KO-1 podocytes showed an increased recruitment of NUP107 and mAb414 detectable NPCs to the nuclear envelope (Figure 2e,f). To further test the concept of NUP107 degradation, as a contributing factor to the *NUP133* KO phenotype, we tried to generate *NUP107* KO podocytes. However, despite several attempts, and using different gRNAs, we were not able to establish a stable monoclonal *NUP107* KO cell line. These observations are in line with previous reports and point to a potentially essential role of NUP107 for cell survival [11]. To bypass this experimental limitation, we created a doxycycline-inducible, CRISPR/Cas9-based *NUP107* KO podocyte cell line (Figure 2e). Doxycycline induction resulted in a subacute loss of NUP107 and mosaic population of KOs. Analyzing this heterogeneous model revealed a severe reduction of NPCs and almost complete absence of NUP133 from the nuclear envelope in cells with identified NUP107 loss (Figure 2f). These findings indicate a mutual, and probably inseparable, role of NUP133 and NUP107 interactions in the pathogenesis of hereditary nucleoporopathies.

### 3.3. Loss of NUP133 Remodels the Transcriptome of Specific Regulatory Pathways

**Figure 3:**
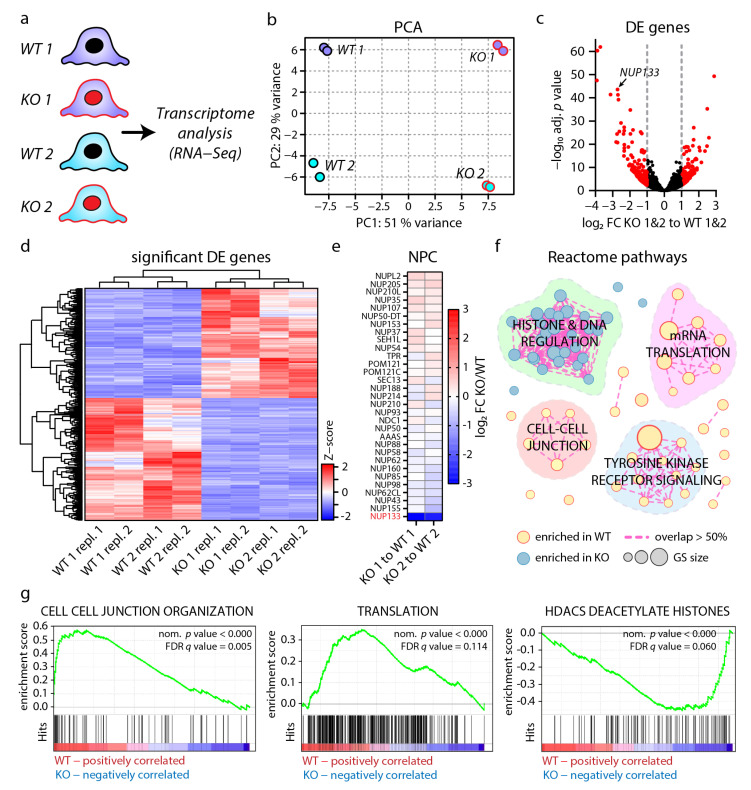
Loss of NUP133 remodels the transcriptome of specific regulatory pathways. (**a**) Schematic of the experimental design for transcriptome analysis (RNA sequencing). Two replicates per *NUP133* genotype and clone were sequenced. (**b**) Principle component analysis (PCA) of transcriptome data (color-coding of dots/clones as in panel). (**a**) Variance of principle component 1 (PC1), mainly depending on the *NUP133* genotype on both monoclonal backgrounds. (**c**) Volcano plot of combined differential expression (DE) analysis for *NUP133* KO-1&2 versus WT-1&2 clones (all 4×WT and 4×KO clones were pooled for statistical analysis). Red dots indicate 324 significant (adjusted *p*-value < 0.05 and log2 fold-change (FC) > (abs. ± 1)) regulated transcripts. (**d**) Heat map showing Z-scores for these 324 significantly regulated transcripts, as well as unsupervised grouping for all analyzed samples. (**e**) Expression levels of NUP transcripts are not significantly altered (adj. *p*-value < 0.05 and log2 FC > (abs. ±1)), with the exception of *NUP133*. (**f**) Gene set enrichment analysis (GSEA) for altered reactome pathways. Significantly enriched gene sets (GS) (nominal *p*-value < 0.01 and FRD *q*-value < 0.25) were clustered to a network, based on their overlap of mapped genes. (**g**) Representative GSEA plots of enriched reactome pathway gene sets are shown.

The NPC is essentially involved in transcriptional regulation, not only by nucleocytoplasmic transport, but also as a scaffold for direct regulation of gene activity [16,17,18,19]. To dissect the impact of NUP133 loss-of-function on regulation of gene activity, we analyzed the transcriptome of *NUP133* KO-1&2 and WT-1&2 podocyte cell lines by employing bulk RNA-sequencing (Figure 3a). This analysis demonstrated a remarkably large set of 2069 differentially expressed genes (adjusted *p*-value < 0.05), clearly separating KO and WT cells on both monoclonal backgrounds (Figure 3b, Supplementary Dataset S1). Moreover, 324 of these significantly regulated genes exhibited more than two-fold differential expression levels (Figure 3c,d and Figure S3 and Dataset S1).

Based on the impact of loss of NUP133 on NPC assembly and *NUP107* expression levels (Figure 1 and Figure 2), we examined the expression level of NUP coding genes in this transcriptome dataset (Figure 3e). With the exception for *NUP133*, no overt regulation of NUP transcription was detectable, further substantiating the concept of altered protein dynamics as the essential underlying mechanism for observed NPC phenotypes.

To obtain a global overview of the NUP133-dependent transcriptional landscape, gene set enrichment analysis (GSEA) was performed by utilizing the gene sets curated by the gene ontology and reactome resource (Dataset S1). Further network analysis of altered reactome gene sets revealed four functional clusters; three clusters relatively enriched in WT cells, encompassing mRNA translation, cell-cell junctions, and tyrosine kinase receptor signaling, as well as one cluster enriched in KO cells encompassing histone-mediated DNA regulation (Figure 3f,g and Figure S3 and Dataset S1).

### 3.4. The Podocyte-Specific Transcriptome Is Disturbed by Loss of NUP133

**Figure 4:**
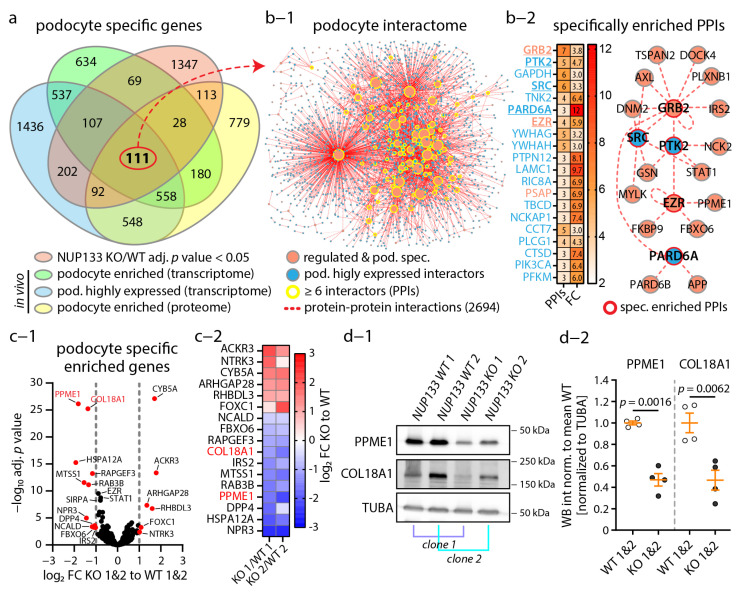
Loss of NUP133 disturbs the podocyte-specific transcriptome. (**a**) Venn diagram analysis of NUP133-dependent differentially expressed genes (adj. *p*-value < 0.05), and murine in vivo podocyte transcriptome- and proteome-based expression data reveals a highly podocyte-specific set of 111 genes that were altered by NUP133. (**b-1**) Protein interaction network analysis for encoded proteins of these 111 genes suggests disruption of a large group of protein complexes expressed in podocytes in vivo. Network showing the altered podocyte interactome (nodes indicate proteins and dashed lines indicate protein–protein interactions (PPIs); node size indicates number of PPIs; NUP133 regulated genes (encoded proteins) and podocyte interactors are color-coded as indicated). (**b-2**) Enrichment analysis for podocyte PPI partners revealed enrichment of an interconnected group of cytoskeletal regulators. Overrepresented PPI partners are presented as a heat map (**b-2**), and a representative interaction network of these proteins is shown (nodes/proteins are color-coded as in graph (**b-1**); the heat map is color-coded as indicated for PPI number and enrichment (FC—fold change)). (**c-1**) Further analysis of in vivo podocyte-specific expressed genes, mapped by differential expression analysis of *NUP133* WT and KO podocytes, identified PPME1 and COL18A1 as downregulated genes. Volcano plot shows NUP133-dependent regulation of in vivo podocyte-specific enriched genes (red dots indicate NUP133-dependent regulated genes (log2 FC > (abs. ± 1) and adj. *p*-value < 0.05)). (**c-2**) Heat map shows the regulation of the gene transcripts identified in c-1 on individual monoclonal podocyte backgrounds. (**d-1**) Western blot analysis showed PPME1 and COL18A1 downregulation in *NUP133* KO podocytes on the protein level (square brackets indicate matching monoclonal backgrounds, with different absolute expression levels, but similar relative downregulation for these proteins). Alpha-tubulin (TUBA) was used as loading control. (**d-2**) PPME-to-TUBA or COL18A1-to-TUBA western blot band density of two independent replicates was analyzed (WT-1&WT-2 and KO-1&KO-2 clones were pooled for statistical analysis and graphical presentation (scatterplot)).

Based on this initial analysis (Figure 3), we aimed to examine the podocyte-specific implications of this transcriptome. Here, we setup an in silico approach, where we merged the in vitro NUP133 transcriptomes with available expression datasets mapping the podocyte-specific proteomes and transcriptomes in vivo (Dataset S1). This approach yielded a set of 111 highly podocyte-specifically expressed genes, significantly altered by the loss of NUP133 (Figure 4a and Dataset S1).

Next, we used the proteins encoded by these genes and BioGrid database to generate a protein–protein interaction (PPI) network of podocyte-specific expressed interactors (Figure 4b-1,b-2). This network revealed a large set of 2694 PPIs and protein complexes that were potentially affected in *NUP133* KO podocytes. We analyzed this network for specifically overrepresented interactors in podocytes, in order to further dissect this large group of potential interaction partners (Figure 4b and Dataset S1). This in-depth analysis resulted in a network of interactors with known relevance for podocyte biology, such as GRB2, PTK2, SRC, PARD6A, and EZR. Interestingly, many of these interactors are involved in, or directly linked to, cytoskeleton regulation. Further analysis of podocyte-specific gene transcripts identified a set of 17 genes, which were more than two-fold differentially regulated (Figure 4c-1,c-2). Within this set of genes, PPME1 and COL18A1 were the most significantly downregulated gene products, not only in mRNA, but also on the protein level (Figure 4d-1,d-2). Together, these data implicate a versatile and profound impact of *NUP133* loss-of-function on the podocyte transcriptome involving many genes and gene products known for their essential role in glomerular health and disease.

### 3.5. Causative NUP133 Variants Do Not Significantly Alter Transcriptome Composition

**Figure 5:**
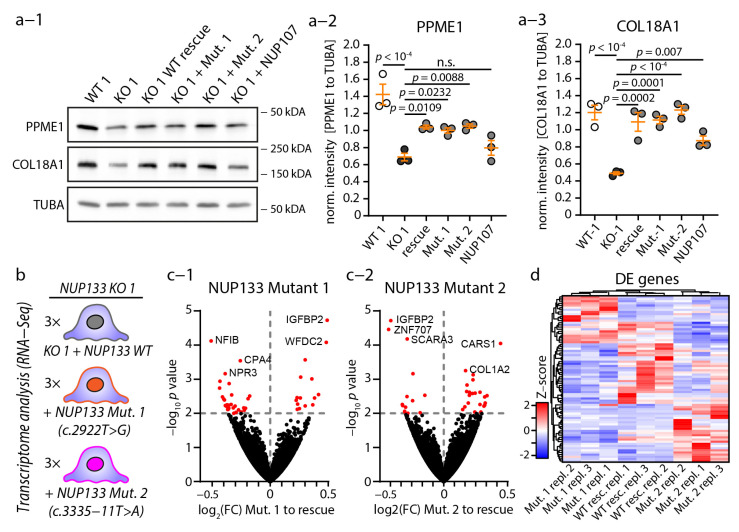
NUP133 variants do not significantly alter transcriptome composition. (**a-1**,**a-2**,**a-3**) Western blot analysis of NUP133 KO, WT, and mutant cell lines for PPME1 and COL18A1. Alpha-tubulin (TUBA) was used as loading control and three independent replicates were analyzed (scatterplots; n. s.—non-significant). (**b**) Schematic of the experimental setup for transcriptome analysis of *NUP133* KO-1 podocytes, expressing either WT or mutation carrying *NUP133* cDNAs. Three replicates, per genotype and clone, were sequenced, and mutant variants were compared to *NUP133* WT. (**c-1**,**c-2**) Volcano plot of differential expression analysis for NUP133 Mutant-1 or Mutant-2 versus *NUP133* WT (rescue) podocytes. Red dots indicate 45 differential expressed gene transcripts for Mutant-1 (**c-1**), with 37 for Mutant-2 ((**c-2**); *p*-value < 0.01). (**d**) Heat map showing Z-scores for these 77 by at least one mutant variant regulated transcripts, as well as unsupervised grouping of analyzed samples.

After characterizing transcriptional changes in *NUP133* KO podocytes, we decided to examine causative *NUP133* variants for similar regulatory events. Given the robust regulation of COL18A1 and PPME1 in KO cells, we analyzed the protein levels of these candidates. However, mutant variants of *NUP133* were able to rescue the KO effects and showed no difference to cells rescued by the wild-type variant of *NUP133* (Figure 5a-1,a-2,a-3).

Therefore, we performed a comparative transcriptome analysis of *NUP 133* KO-1 podocytes expressing *NUP133* WT or Mutant-1 or Mutant-2 variants, in order to dissect the mutant-specific effects on transcriptional regulation (Figure 5b). Unexpectedly, no major and significant alterations were detectable by differential expression analysis (Dataset S2—adjusted *p*-value < 0.05). In order to describe only minute differences, we adapted the stringency of applied cut-off values (non-adjusted *p*-values *p* < 0.01), resulting in the detection of a set of 45 genes differentiating Mutant-1 form WT and 37 genes differentiating Mutant-2 from WT *NUP133* (Figure 5c,d and Dataset S2). Individual genes (e.g., NPR3) exhibited at least regulatory trends, according to the observed changes in *NUP133* KO cells. In summary, these observations, on the transcriptome level, argue against a strict loss- or gain-of-function mechanism, related to SRNS-causative *NUP133* variants.

### 3.6. Loss of NUP133 Disrupts Dynamic Cytoskeleton Regulation in Podocytes

**Figure 6:**
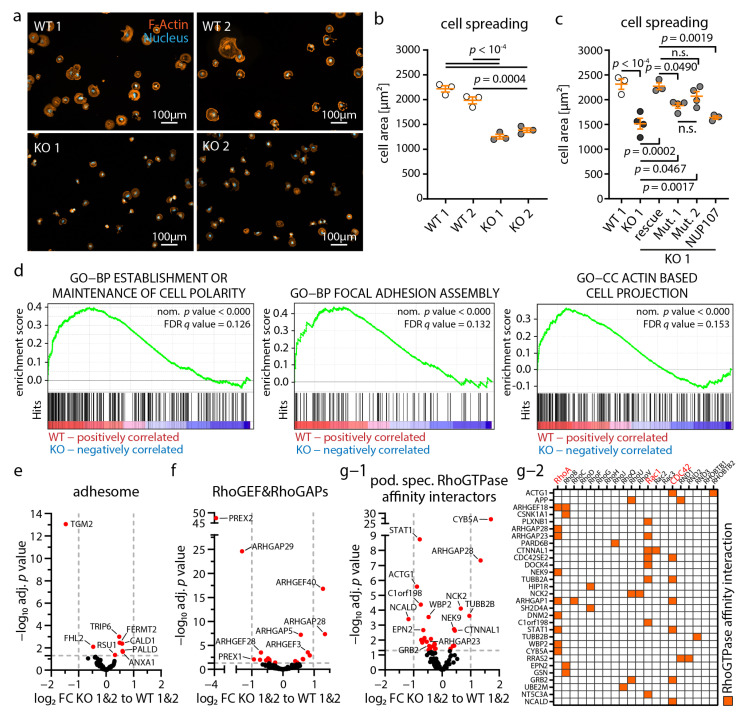
Loss of NUP133 disturbs dynamic cytoskeleton regulation in podocytes. (**a**–**c**) Cell spreading analysis of *NUP133* WT, KO, and mutant cell lines showed reduced spreading capacity of KO and mutant podocytes (F-actin was stained by phalloidin and nuclei by Hoechst 33342). Mean cell areas, from at least three independent experiments per genotype and clone, are shown (scatterplots indicate mean values of independent experiments, at least 100 cells per replicate were analyzed; n. s.—non-significant). (**d**) Representative GSEA plots of enriched gene ontology (GO) gene sets, correlating with cell adhesion, protrusion, and polarization. (**e**–**g-2**) Volcano plots of differential expression analysis of *NUP133* KO-1&2 versus WT-1&2 clones. Red dots indicate significantly regulated transcripts (adjusted *p*-value < 0.05). Analysis for consensus adhesome proteins, RhoGAP&RhoGEFs, and podocyte-specific small RhoGTPase affinity interactors demonstrated slight regulation of adhesome genes, but marked regulation of small RhoGTPase signaling pathways. (**g-2**) Heat map of altered RhoGTPase affinity interactors identified in (**g-1**), as well as the corresponding RhoGTPases demonstrates regulation of RhoA, Rac1, and Cdc42 signaling pathways.

Given the extensive transcriptomic shift in our *NUP133* KO model, we were wondering how such regulatory events might translate into impaired podocyte function (Figure 3). Notably, we observed marked alterations and protein complex disruptions of cytoskeleton-associated proteins, upon the loss of NUP133 (Figure 4). Due to the essential role of the podocyte cytoskeleton for the establishment and maintenance of the glomerular filtration barrier, we performed cell spreading assays as a surrogate marker for dynamic actin cytoskeleton adaptation, such as integrin adhesion turnover, protrusion formation, and small RhoGTPase signaling (Figure 6a–c). These experiments demonstrated a reduced spreading capacity of the respective *NUP133* KO and mutant podocytes. In line with our functional observations, gene sets representing these processes were significantly enriched in WT, compared to KO cells (Figure 6d). Interestingly, the composition within these gene sets indicated predominant alterations of a rather regulatory nature, rather than of structural components (Dataset S1). Spreading KO podocytes showed a less organized actin cytoskeleton and features, such as cell membrane ruffling, but no obvious alteration of cell size, morphology, actin cytoskeleton, or integrin adhesion complex (IAC) structures at steady-state conditions (Figure S4). The complex process of cellular spreading includes different aspects of cytoskeletal remodeling, involving adhesion site maturation and intricate intracellular signaling pathways. To further dissect potential underlying pathways involved in the impaired spreading capacity of *NUP133* KO cells, we evaluated the respective gene sets. Detailed analysis of consensus integrin adhesome components showed only mild differential expression of these genes (Figure 6e). However, RhoGEFs and RhoGAPs, as master regulators of small RhoGTPases mediating actin cytoskeleton signaling, exhibited significant alterations [50,51]. Differential expression analysis revealed 19 significantly (adj. *p*-value < 0.05) regulated RhoGEFs and RhoGAPs, as well as four significantly regulated small RhoGTPases (RHOD, RHOBTB1, RHOF, and RHOJ) (Figure 6f and Dataset S1). Further integration of a recently published network of podocyte-specific RhoGTPase proximity/affinity interactors revealed 30 podocyte-specific (and 191 non podocyte-specific) differentially expressed genes in *NUP133* KO cells (Figure 6g and Dataset S1). Mapping the RhoGTPase proximity interactors of these 30 podocyte-specific expressed proteins showed an enrichment of RhoA, Rac1, and CDC42 interactors (Figure 6g-1,g-2). In summary, loss of NUP133 appears to disrupt the RhoGTPase signaling networks on a transcriptomic level, thereby impairing the dynamic generation of cell protrusions in podocytes.

## 4. Discussion

Understanding the intricate mechanisms leading to podocyte-specific manifestations of nucleoporopathies might provide novel insights in podocyte biology and potential therapeutic approaches for a given SRNS disease conditions. Here, we generated an in vitro model for NUP133-associated podocytopathy. We employed *NUP133* loss-of-function via CRISPR/Cas9-mediated generation of KO cells, as well as SRNS-causing mutations by re-expression of *NUP133* c.2922T>G (p.Ser974Arg) and *NUP133* c.3335-11T>A (p.1111_1112insValPheIle) variants in human podocytes (Figure 1 and Figure 2).

Podocytes are highly specialized epithelial cells, relying on a tightly controlled cell type-specific transcriptome and proteome to regulate, e.g., adhesion, polarity, and protrusion formation, in order to maintain the integrity of the kidney filtration barrier [28,30,31,42,52,53,54]. Beyond nucleocytoplasmic transport, NPCs and NUPs are involved in gene expression via chromatin organization, direct transcriptional regulation, and as a scaffold for the transcriptional machinery [16,17,18,19,55]. Recently, a study analyzing the embryonic development of *Nup133*-deficient mice demonstrated the essential requirement of NUP133 for terminal differentiation of neurons and embryonic stem cells [56]. Based on these previous studies, we speculated that *NUP133*-associated nucleoporopathies might influence the expression levels of specific genes and, thereby, podocyte functionality. Indeed, transcriptome analysis of *NUP133* KO cells revealed extensive expressional alterations, involving many podocyte-specific genes and subcellular machineries (Figure 3 and Figure 4). These findings are exemplified by the downregulation of COL18A1 and PP2A master regulator PPME1, two proteins with essential functions in podocytes [57,58].

Mechanistically, the loss of NUP133 leads to a defective and decreased assembly of NPCs in the nuclear envelope (Figure 1 and Figure 2). Therefore, the observed phenotypes might be attributed to disturbed function of NPC and/or individual NUPs. Interestingly, a recent study highlighted the different roles of Nup107 and Nup93 for gene regulation [59]. While Nup107 targets active genes, Nup93 is involved in the silencing of Polycomb target genes. Intriguingly, NUPs, NUP107, and NUP93 carry hereditary mutations causing SRNS [10,11,13,14]. Moreover, NUP107 is a direct and essential interactor of NUP133 in the Y-complex [46]. Analyzing NUP107 in *NUP133* KO cells revealed normal *NUP107* transcription, but reduced protein levels, indicating elevated protein turnover, as a consequence of Y-complex destabilization (Figure 1, Figure 2 and Figure 3). Furthermore, SRNS-causing mutations of *NUP133* and *NUP107* have been shown to weaken the protein–protein interaction of these two proteins [11,12,13]. Moreover, mutations in *NUP107* and *NUP133* cause very similar phenotypes: both genes carry mutations that cause isolated podocytopathy (SRNS) starting in early life years or SRNS with additional neuronal disorders [11,12,13]. These findings implicate the closely related or mechanistically connected disease mechanism of *NUP107* and *NUP133* mutations. This hypothesis was further substantiated by the analysis of *NUP107* KO podocytes, revealing similar NPC formation defects, as in *NUP133* KO cells (Figure 2). Given the reported role of NUP107 for gene regulation, as well as the observation that we, and others, were not able to generate a stable *NUP107* KO podocyte cell line (likely due to lethality of this line, in contrast to *NUP133*), it can be speculated that NUP133-dependent phenotypes might be partially, or even primarily, mediated by NUP107 [11]. However, expression of *NUP107* in *NUP133* KO podocytes led to only partially restored COL18A1 levels and showed no impact on PPME1 expression or cell spreading defects (Figure 5 and Figure 6). Additionally, NUP107 is still partially recruited to the nuclear envelope in *NUP133* KO cells (Figure 2), likely via binding to other Y-complex nucleoporins [60]. Other studies demonstrated that NUP133 can localize to NPCs, independently of NUP107 [61]. Therefore, degradation of NUP107 contributes to certain aspects of the NUP133 phenotype, but structural disruption of the NUP107–NUP133 interaction and the Y-complex appears to be more relevant, as implicated by the several mutations of *NUP107* and *NUP133* impairing (but not abolishing) this interaction (Figure S2) [11,12,13].

Along this concept, SRNS caused by Y-complex nucleoporopathies probably translates into decreased assembly, clustering, or structural weakening of the whole NPC. In line with this, pioneering studies, first describing NUP133 in Saccharomyces, reported the altered clustering of NPCs to distinct foci within the nuclear envelope [62,63]. However, we observed decreased NPC localization within the nuclear envelope and cytoplasmic enrichment of nucleoporins, but no distinct change in nuclear localization of NPCs (Figure 1 and Figure 2). This difference can be attributed to the evolutionary differences between mammalian and yeast NPC, but the underlying mechanisms might be similar. Therefore, it might be promising to investigate NPC clustering and structure within the nuclear envelope in detail in *NUP133* KO and mutant cell lines, using super resolution or electron microscopy, in future studies. In summary, observed phenotypes might be attributed to multiple alterations of different NUPs and, thereby, explain the vast and complex transcriptional differences observed in *NUP133* KO cells.

As an alternative mechanism to this “structural loss-of-function” hypothesis, gene expression may be influenced as a direct consequence of causative mutations. However, analyzing the transcriptome of *NUP133* mutant podocytes revealed no significant changes in expressed genes, largely excluding a direct loss- or gain-of-function mechanism (Figure 5). However, *NUP133* mutant podocytes exhibited mild defects in the localization of NUP133 and NUP107 to the nuclear envelope, as well as spreading defects (Figure 3, Figure 5, Figure 6 and Figure S1). Such slight impairments might only have relevant effects in sensitive cell types, which depend on the tight expressional control of a given type of signaling pathways and/or have low functional reserves, e.g., NUP133 [15,16]. This concept is supported by a recent study in zebrafish, demonstrating that moderate *Nup133* deficiency is sufficient to induce podocyte damage, but does not translate into a developmental kidney phenotype [64]. Moreover, reduced NUP133 expression levels have been reported in patient-derived cell lines before [12]. Of note, our mutant model does not test for the consequences of increased NUP133 turnover at endogenous expression levels. Therefore, a decrease of NUP133 protein levels might be the primary consequence of mutant variants, finally resulting in a knockdown or partial loss-of-function phenotype in susceptible cell types.

In podocytes, one such susceptible and essential signaling pathway is small RhoGTPase signaling [31,65,66,67]. Indeed, activation of the small RhoGTPase Cdc42 has been described as a common consequence of the loss of NUP85, NUP107, and NUP133 in podocytes [11]. Moreover, cell spreading (as a surrogate marker for protrusion generation, actin cytoskeleton, integrin adhesion function, and RhoGTPase signaling) was impaired in *NUP133* KO podocytes (Figure 6). Further analysis, with respect to the expression of small RhoGTPase signaling pathways, revealed altered composition of RhoA, Rac1, and Cdc42 pathways in *NUP133* KO podocytes (Figure 6). Imbalanced RhoGTPase signaling impairs multiple cytoskeleton processes, but, most importantly, the generation of cell protrusions and, thereby, establishment of the complex foot process architecture of podocytes [31,65,68,69]. The exact mechanism of NUP133-dependent regulation of gene expression remains largely unknown, but several nucleoporins, including NUP107 have been identified to interact with transcriptional active chromatin [18,59,70]. This nucleoporin–chromatin binding likely facilitates and stabilizes chromatin organization and, thereby, transcriptional gene regulation. Therefore, the establishment and maintenance of cell type-specific gene expression patterns might be facilitated by nucleoporins, such as the expression of small RhoGTPase signaling pathway genes in podocytes. Based on these findings, two mechanisms for altered gene regulation in *NUP133* KO cells can be hypothesized. First, the (partial) disruption of the NPC impairs abundance, localization, and function of many NUPs, with known effects on gene expression, e.g., as implicated by the effects of *NUP107* or *NUP133* knockdown on the chromatin binding of FG-NUPs [71]. Second, the NUP107 chromatin binding complex is likely impaired by reduced *NUP107* expression levels in *NUP133* KO cells [59]. Moreover, NUP133 might be a component of the NUP107 chromatin binding complex, given the well-documented interaction of these two nucleoporins [46]. Intriguingly, both mechanisms involve NUP107, or effects that had been shown to be exerted by NUP107, by a similar mechanism, again showing the close interdependency of NUP133 and NUP107 mediated phenotypes, implicating similar mechanisms of *NUP133* and *NUP107* mutant variants in SRNS.

## 5. Conclusions

In summary, a low resistance to declining NUP133 levels and impaired small RhoGTPase signaling might explain the susceptibility of podocytes for disease manifestation in hereditary nucleoporopathies. Functional impairment of the NUP107–NUP133 interaction might be the primary defect of the SRNS-causing variants of *NUP107* and *NUP133.* Therefore, pathomechanisms-like transcriptome alteration might be similar, or closely related, for both genes. The mild consequences of *NUP133* mutant variants (as observed in cell culture) probably translate into severe phenotypes in more complex model systems, such as organoids, drosophila melanogaster, or mus musculus [72,73,74,75]. Further studies, using these models, might evaluate the therapeutic approaches for targeting small RhoGTPase signaling or the inhibition of proteasomal degradation (e.g., NUP107) [26,76,77].

## Data Availability

The data presented in this study are available in the article or Supplementary Materials. Sequencing data have been deposited in NCBI Gene Expression Omnibus and are accessible via GEO series, accession numbers GSE189703 and GSE189709.

## Supplementary Materials

The following supporting information can be downloaded at: https://www.mdpi.com/article/10.3390/cells11081259/s1, Figure S1: Analysis of *NUP133* KO nuclei, corresponding to Figure 1 and Figure 2; Figure S2: Analysis of the NUP107 to NUP133 protein interaction, corresponding to Figure 2; Figure S3: Analysis of the *NUP133* dependent transcriptome, corresponding to Figure 3 and Figure 6; Figure S4: Analysis of cell spreading and integrin adhesion complexes of *NUP133* KO cells, corresponding to Figure 6; Dataset S1: Transcriptome analysis of *NUP133* KO cells, corresponding to Figure 3; Dataset S2: Transcriptome analysis of *NUP133* Mutant cells, corresponding to Figure 5.
