# Supplementary material for: NUP133 Controls Nuclear Pore Assembly, Transcriptome Composition, and Cytoskeleton Regulation in Podocytes
Source: Cells. 2022 Apr 7;11(8):1259. doi: 10.3390/cells11081259 (PMC9025798; doi:10.3390/cells11081259)
Supplement: Supplementary file 1 [file cells-11-01259-s001.zip › cells-1614276-supplementary/Supplemental Figures.pdf]

Supplemental Figures

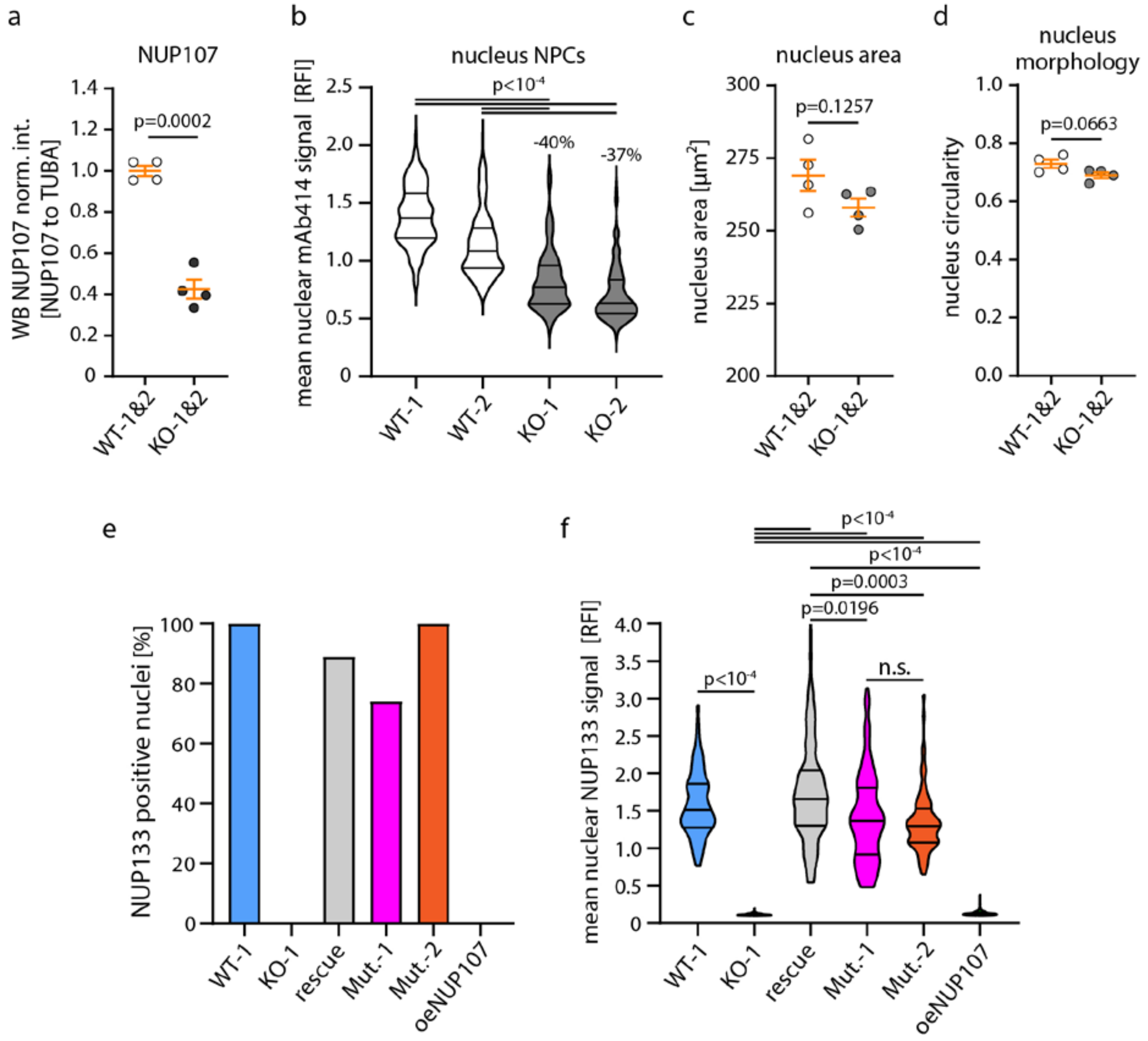

Figure S1

(a) Western blot quantification of NUP107 expression (related to Figure 1c). NUP107 band intensity was analyzed in two independent experiments and normalized mean WT intensity (TUBA was used as loading control; NUP107 intensities were corrected for TUBA signal intensity; data from WT-1 & WT2, as well as KO-1 & KO-2 were pooled for graphical presentation and statistical analysis). (b) Nuclear mAb414 signal intensities were measured and relative fluorescence intensities (RFI) were expressed as violin plots (at least 140 cell per genotype were analyzed; percentages indicate reduction of mean RFI of WT-1 compared to KO-1 and WT-2 to KO-2). (c-d) Analysis of nuclear area and morphology (circularity) showed no significant differences between *NUP133* WT and KO podocytes (scatter dot plots show mean values for WT-1, WT-2, KO-1 and KO-2 clones from two independent experiments; at least 32 nuclei per experiment and clone were analyzed; WT-1&2 and KO-1&2 values were pooled for statistical comparison). (e-f) Corresponding immunofluorescence analysis to Figure 2e. Cell nuclei of indicated cell lines were quantified for NUP133 positivity. (e) The percentage of NUP133 positive nuclei is presented. (f) Nuclear NUP133 staining signals (only of NUP133 positive cells/nuclei for rescue, Mutant-1 and Mutant-2 cell lines) were measured and relative fluorescence intensities (RFI) were expressed as violin plot (at least 147 cell per genotype were analyzed; n.s. - non-significant).

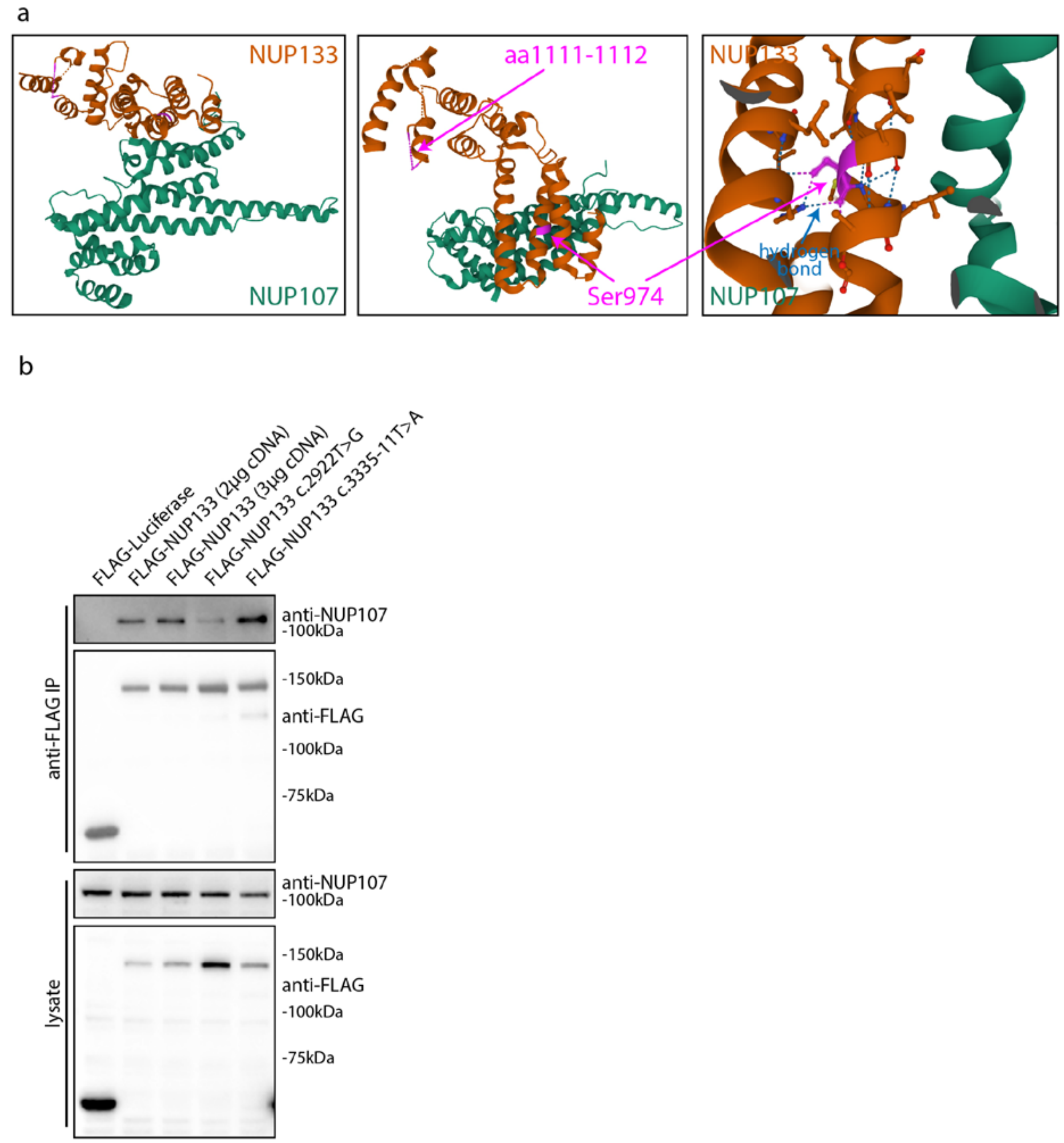

Figure S2

(a) 3D structure of the human nucleoporin NUP107 (green) – NUP133 (orange) interaction complex (PDB DOI: 10.2210/pdb3CQC/pdb; model shows NUP107 aa667 to aa924 and NUP133 aa932 to aa1156; aa – amino acid). Positions of mutant variants are color coded in purple. *NUP133* mutation variant "-1" - p.Ser974Arg (c.2922T>G) - replacing Ser974 by Arg and is located in a helix within the NUP107 – NUP133 interaction interface. *NUP133* mutation variant "-2" - p.1111_1112insValPheIle (c.3335-11T>A) - causing the insertion of an 9bp intronic sequence (3 amino acids: Val-Phe-Ile between NUP133 aa1111 and aa1112) from the splice site between exons 25 and 26 of the *NUP133* gene. Mutant variant-2 is located in an unmodeled residue c-terminally of the NUP107 – NUP133 interaction interface. (b) Protein interaction analysis for NUP133 variants with NUP107 by immunoprecipitation of FLAG-tagged NUP133 or Luciferase as negative control. Balancing protein expression for NUP133 pulldown levels revealed impaired complex formation of mutant variant-1 with NUP107 but even increased pulldown of NUP107 by mutant variant-2 (two different amounts of *NUP133* WT cDNA were expressed as reference as indicated).

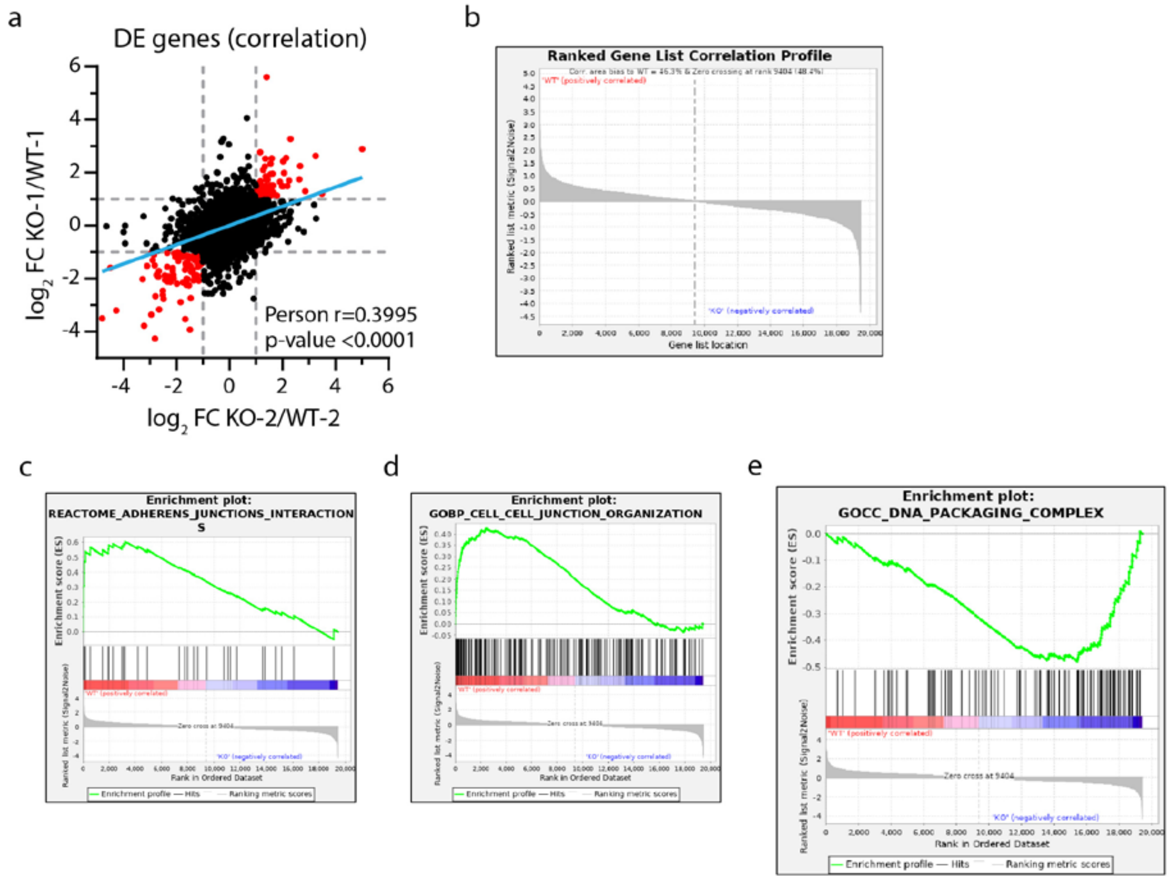

Figure S3

(a) Transcriptome analysis corresponding to Figure 3c. Correlation analysis (scatterplot) of monoclonal *NUP133* KO/WT background "1" versus "2" (regression line is shown in blue; person correlation was calculated; red dots indicate significant regulated transcripts with correlating $\log_2$ FC > (abs. ±1) for both cellular backgrounds). (d-g) GSEA plots related to and extending enrichment analysis presented in Figure 3g and Figure 6d.

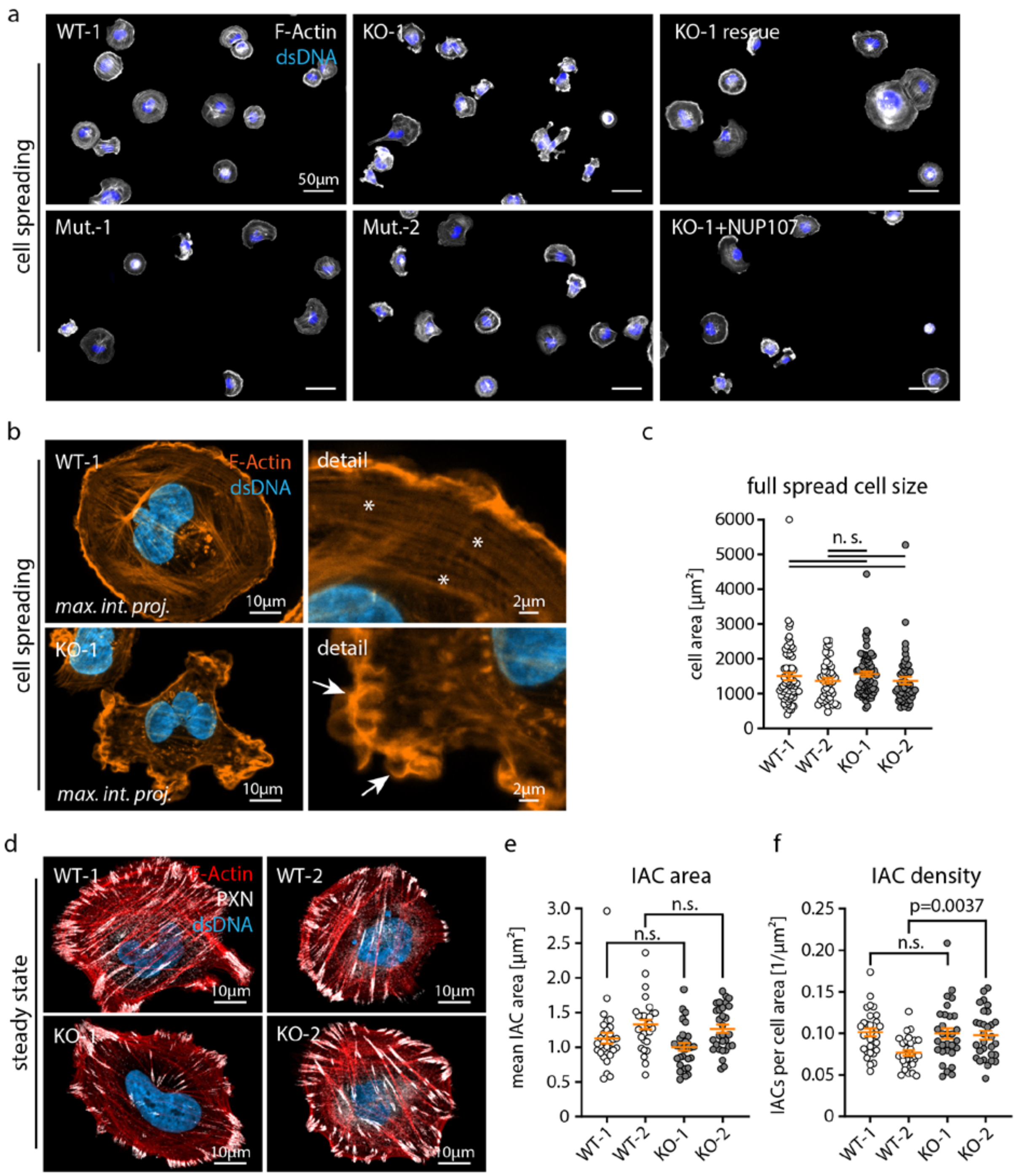

Figure S4

(a) Representative overview images of spreading cells related to analysis in Figure 6c. Indicated cell lines were seeded on collagen IV for 30 minutes and stained for F-Actin by Phalloidin and cell nuclei by Hoechst 33342. (b) Representative immunofluorescence analysis related to Figure 6a. Cells were stained for F-Actin (Phalloidin) and nuclei (Hoechst 33342). White asterisks indicate transversal F-Actin arcs in WT cells. White arrows indicate membrane ruffling and blebbing of the cell edge in *NUP133* KO cells. Maximum intensity projections of z-stacks are shown. (c) Quantification of cell area of fully spread cells (24h after seeding) revealed no obvious alteration of cell size (n. s. – non-significant; 45 to 70 cells per genotype were analyzed). (d-e) Analysis of integrin adhesion complexes (IAC) at steady state conditions (24h after seeding) showed no congruent alteration of IAC area or density (n. s. – non-significant; 30 cells per genotype were analyzed). IACs were labeled by Paxillin (PXN), F-Actin by Phalloidin and nuclei by Hoechst 33342 immunofluorescence staining.
